# Supplementary material for: Presence of Mycoplasma bovis in Bulk Tank Milk and Associated Risk Factor Analysis in Serbian Dairy Farms
Source: Pathogens. 2024 Apr 6;13(4):302. doi: 10.3390/pathogens13040302 (PMC11054648; doi:10.3390/pathogens13040302)
Supplement: Supplementary file 1 [file pathogens-13-00302-s001.zip › pathogens-2914442-supplementary.pdf]

Study: *Mycoplasma bovis* in bulk tank milk - risk factor analysis in Serbia

Researcher name: Milan Ninković

Name Farm:

Location:

Date:

Questionnaire

Number of cows at the farm	---------------
Milk yield	---------------
Average of parity on the farm	--------------
Number of cows in terms of BTM	--------------
Presence of *M. bovis* mastitis	YES/NO
Breed	HF/SIM
Presence of bacterial mastitis	YES/NO
Do you use of disinfection before milking	YES/NO
Do you use of disinfection after milking	YES/NO
Type of farm	family farm/corporate farm
Type of milking	machine/manual
Type of holding	tie stall/free stall
Presence of overcrowding of stall	YES/NO
Presence of inadequate ambition condition on farm	YES/NO

……………………………………………

Signature
